# Letter to the editor regarding “Atlantoaxial dislocation due to os odontoideum in patients with Down’s syndrome: literature review and case reports”

**DOI:** 10.1007/s00381-020-04886-y

**Published:** 2020-09-17

**Authors:** Fraser C. Henderson, Clair A. Francomano, Peter C. Rowe

**Affiliations:** 1 Department Neurosurgery, University of Maryland Capital Region Health Center, Cheverly, MD USA; 2 Metropolitan Neurosurgery Group LLC, 1010 Wayne Avenue, Suite 420, Silver Spring, MD 20910 USA; 3 Department of Medical and Molecular Genetics, Indiana University School of Medicine, 975 W. Walnut Street, IB 130, Indianapolis, IN 46202 USA; 4 Department of Pediatrics, Johns Hopkins University School of Medicine, 200 N. Wolfe Street, #2077, Baltimore, MD 21287 USA

Dear Editor:

We have read with interest the excellent review of Down syndrome by Sergeenko et al. [1], in which they bring to attention the frequent occurrence of atlantoaxial instability (AAI) in Down syndrome (DS), the importance of directed radiological assessment, and the association of AAI with os odontoideum, ligamentous laxity, low bone mineral density, low muscle tone, and excessive joint flexibility.

We would like to draw a parallel with another set of genetic disorders, which are characterized by ligamentous laxity, excessive joint laxity, and frequently, low bone density and also have a higher than expected risk of AAI: the hereditary connective tissue disorders (HCTD), including Ehlers-Danlos, Loeys-Dietz, Marfan, and Morquio syndromes [2].

In both DS and the HCTD, the clinical diagnosis of AAI can be challenging because of the many comorbid conditions and the musculoskeletal symptoms caused by the generalized joint hypermobility. In both DS and the HCTD, AAI usually manifests in childhood and adolescence (when there is greatest ligamentous laxity) or following minor trauma, and neurological deterioration then occurs over several years. In DS, AAI may cause quadriparesis or quadriplegia with neck flexion, whereas in HCTD, AAI causes severe headache, visual symptoms, syncope or pre-syncope, dysesthesias, nausea and tinnitus, hyperreflexia, and sensory changes due to rotational instability between C1 and C2 [3, 4].

**Fig. 1:**
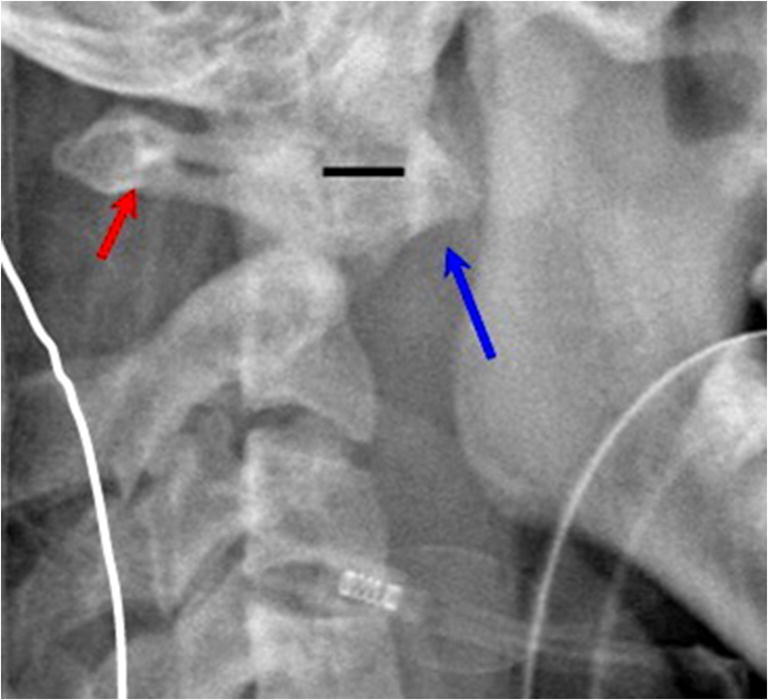
Lateral cervical spine x-ray demonstrates atlantoaxial instability (AAI) in Down syndrome. In this case, mild flexion of the neck demonstrates a widened atlanto-dental interval (solid bar) between the anterior tubercle of C1 (long arrow) and the odontoid process that exceeds the pathological threshold in the adult of 3 mm. The spinal canal (small arrow) is substantially diminished and results in spinal cord compression

**Fig. 2:**
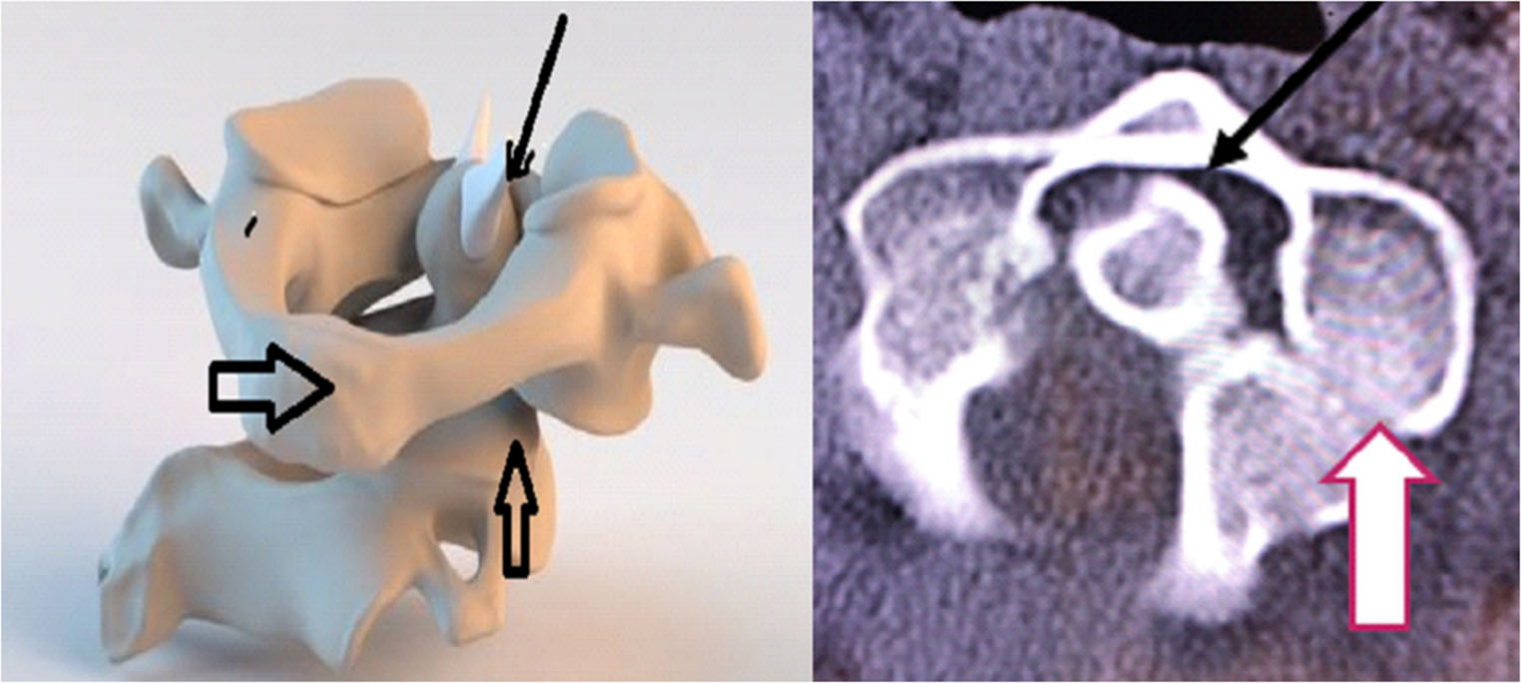
The model in the left image demonstrates the rotary subluxation (AAI Fielding type 1) that occurs with the hereditary connective tissue disorders. With the rotation of the C1 ring (large black arrow), the atlanto-dental interval (small solid arrow) remains normal (< 3 mm), but the facet joint (vertical arrow) is subluxed with more than 80% loss of facet overlap, and the angular displacement between C1 and C2 exceeds the pathological threshold of 41 degrees. On the right, the cervical spine CT (axial view through the interface of C1 and C2) shows the loss of facet overlap (vertical white arrow), but normal atlanto-dental interval (black arrow), representing a rotary subluxation, Fielding Type 1

In DS, screening for AAI has not been reliable, and imaging studies do not correlate well with the risk of myelopathy. Similarly, standard imaging studies fail to show the rotational instability in the HCTD. In these conditions, diagnosis of AAI requires dynamic imaging. In DS, AAI results from incompetence of the transverse odontoid ligament, demonstrated by flexion and extension imaging of the cervical spine (Fig. 1). In HCTD, AAI is caused by incompetence of the alar ligaments, demonstrated on rotational CT by excessive angular displacement between the atlas and the axis (Fig. 2) or by excessive translation on lateral tilt of the neck [4, 5].

Symptoms of the cervical medullary syndrome and tenderness over the C1–C2 joint should prompt suspicion of AAI. Directed neurological exam and imaging are necessary to diagnose clinically significant AAI in these populations [2, 4]. We concur with Sergeenko et al. that posterior stabilization and fusion are reserved only for patients who fail a thorough course of non-operative management.
